# Recent advances in DDAH1 inhibitor design and discovery: insights from structure–activity relationships and X-ray crystal structures

**DOI:** 10.1039/d3ra08210e

**Published:** 2024-03-22

**Authors:** Anthony J. Doman, Michael V. Perkins, Sara Tommasi, Arduino A. Mangoni, Pramod C. Nair

**Affiliations:** a Department of Clinical Pharmacology, Flinders Medical Centre, Southern Adelaide Local Health Network Adelaide Australia; b Discipline of Clinical Pharmacology, College of Medicine and Public Health, Flinders Medical Centre, Flinders University Adelaide Australia pramod.nair@flinders.edu.au +61-8-82043155; c College of Science and Engineering, Flinders University Adelaide Australia; d Flinders Health and Medical Research Institute, Flinders University Adelaide Australia; e Cancer Program, South Australian Health and Medical Research Institute (SAHMRI), University of Adelaide Adelaide SA Australia; f Discipline of Medicine, Adelaide Medical School, The University of Adelaide Adelaide SA Australia

## Abstract

Nitric oxide (NO) is an important signalling molecule which modulates several biological and pathological processes. Dimethylarginine dimethylaminohydrolase 1 (DDAH1) plays a key role indirectly regulating NO concentrations in the body. It has been shown that DDAH1 inhibition may be an effective therapeutic strategy in certain pathological states in which excessive NO is produced. In recent years, specific DDAH1 inhibitors have shown promise in suppressing abnormal neovascularization in cancer. However, the available DDAH1 inhibitors lack potency and selectivity and are mostly arginine-based. Further, these inhibitors display unfavourable pharmacokinetics and have not been tested in humans. Thus, the development of potent, selective, and chemically diverse DDAH1 inhibitors is essential. In this review, we examine the structure activity relationships (SARs) and X-ray crystal structures of known DDAH1 inhibitors. Then, we discuss current challenges in the design and development of novel DDAH1 inhibitors and provide future directions for developing potent and chemically diverse compounds.

## Abstract 2

DDAH1 inhibitors with diverse chemical structures are needed for the development of new therapeutics in NO related disorders.

## Graphical abstract

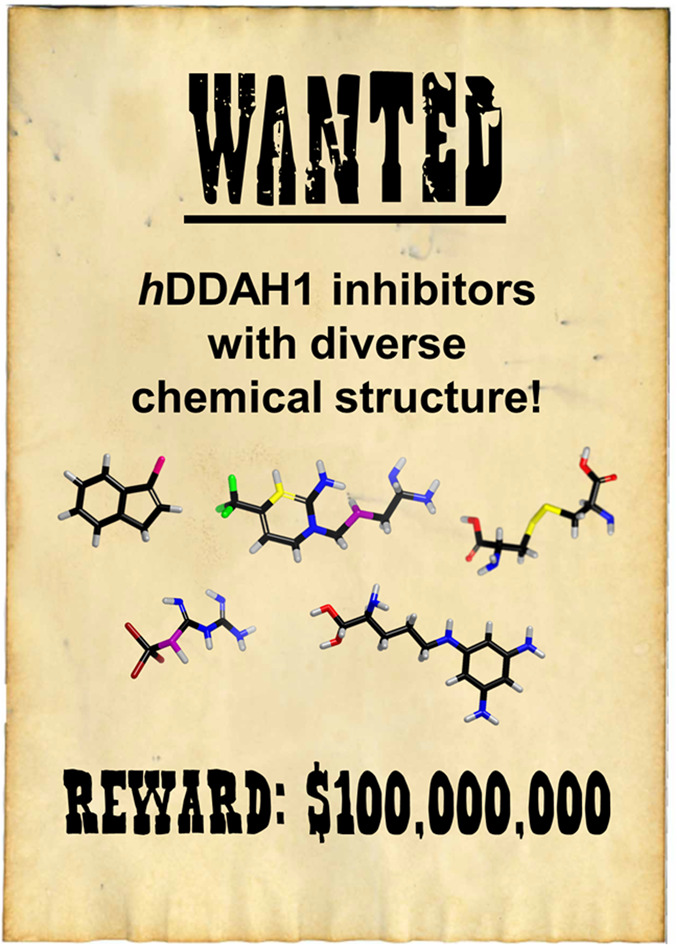

## Introduction

**Fig. 1:**
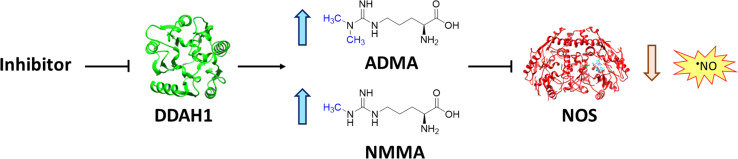
DDAH1 substrates and NOS inhibitors ADMA and NMMA.

Nitric oxide (NO) is a powerful signalling molecule, crucial for the maintenance of physiological processes and immune defences. The homeostatic regulation of neuronal pathways,^1^ vascular tone,^2^ and cytotoxicity in immunity^3^ depends on tight regulation of NO concentrations. Nitric oxide synthase (NOS) is the enzyme responsible for NO synthesis. Excessive NO production is associated with a wide range of pathophysiological states including multiple sclerosis,^4^ sepsis,^5^ Parkinson's disease,^6^ metabolic dysregulation,^7^ and cancer.^8^ The use of NOS inhibitors may directly inhibit NO production, however, simultaneously blocking its beneficial effects. Therefore, in certain disease states, indirect inhibition of NO synthesis may provide better therapeutic outcomes in regulating excess NO.^9^ Dimethylarginine dimethylaminohydrolase 1 (DDAH1) is a key enzyme involved in the metabolism of asymmetric dimethylarginine (ADMA) to l-citrulline and dimethylamine.^9^ ADMA and other substrates of DDAH1 (*e.g.*, L-NMMA or monomethyl-l-arginine) function as potent endogenous NOS inhibitors (Fig. 1). NO concentrations are reduced by the accumulation of ADMA and L-NMMA in cells upon inhibition of DDAH1. In recent years, there has been considerable interest in DDAH1 as a potential target for indirect inhibition of NO synthesis in diseases that produce excessive amounts of NO, such as septic shock,^10^ and aberrant neovascularization,^11^ including cancers such as melanoma,^12^ prostate cancer,^13^ and breast cancer.^11^

Two DDAH isoforms (DDAH1 and DDAH2) have been identified in humans, which share ∼67% protein sequence homology with distinct tissue distribution.^14^ While DDAH1 plays a key role in the NO pathway, it is unclear what role DDAH2 plays. Recently, we demonstrated that DDAH2 does not metabolize ADMA and may have an ADMA independent function.^15^ Given previously known DDAH inhibitors were based on inhibiting ADMA conversion to citrulline, it is likely these compounds inhibit DDAH1 only.

Novel inhibitors targeting DDAH1 have been developed as a result of the crucial importance of fine regulation of the NO pathway (Table 1). For instance, human DDAH1 inhibitor ZST316 (20) was shown to attenuate vasculogenic mimicry and cell migration in triple negative breast cancer (TNBC) cells.^11^ Also, DDAH1 inhibitor DD1E5 inhibits the proliferation of human prostate cancer (PCa) cells and NO production while decreasing the amount of vascular endothelial growth factor (VEGF), thereby inhibiting angiogenesis and tumor progression in mice.^13^

**Table 1:** Evolution of the major DDAH1 inhibitors

Name	Chemical structure	Activity	Author	Year of publication	Reference
4124W (1)	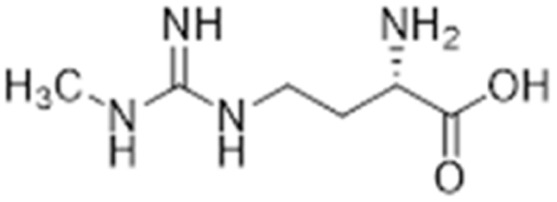	IC_50_ = 1510 μM	MacAllister	1996	16
L-257 (13)	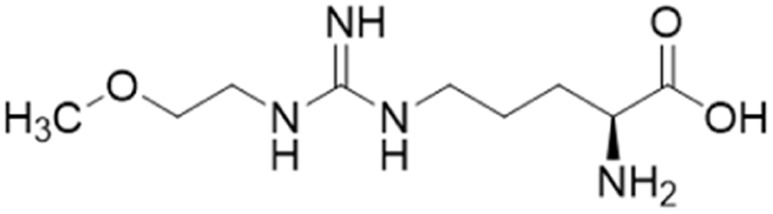	*K*_i_ = 13 μM	Rossiter *et al.*	2005	18
L-VNIO (25)	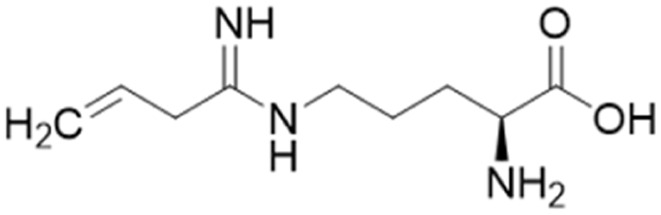	*K*_i_ = 2 μM; IC_50_ = 13 μM	Kotthaus *et al.*	2008	26
4-HNE	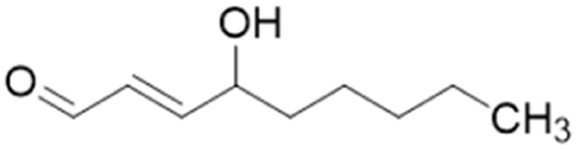	IC_50_ = 50 μM	Forbes *et al.*	2008	46
L-IPO (59)	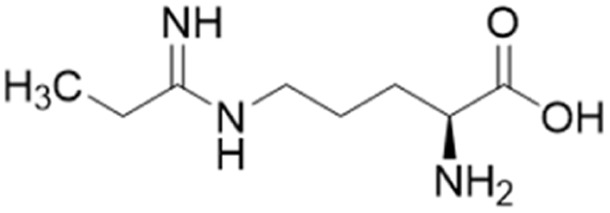	*K*_i_ = 52 μM	Wang *et al.*	2009	32
14^a^ (60)	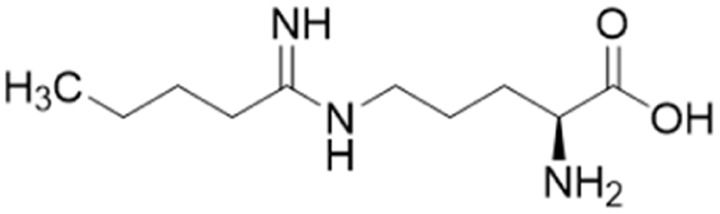	*K*_i_ = 7.5 μM	Wang *et al.*	2009	32
Ebselen	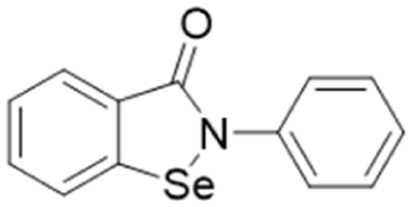	IC_50_ = 0.33 μM	Linsky *et al.*	2011	47
Lansoprazole	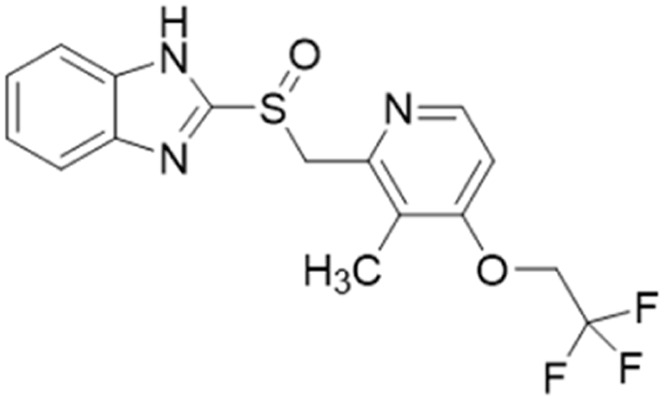	IC_50_ = 51 μM	Ghebremariam	2013	48
PD404182	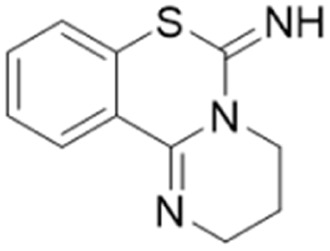	IC_50_ = 9 μM	Ghebremariam	2014	49
ZST316 (20)	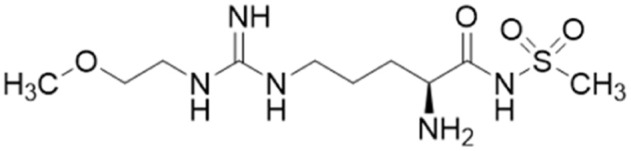	*K*_i_ = 1 μM; IC_50_ = 3 μM	Tommasi *et al.*	2015	22
DD1E5	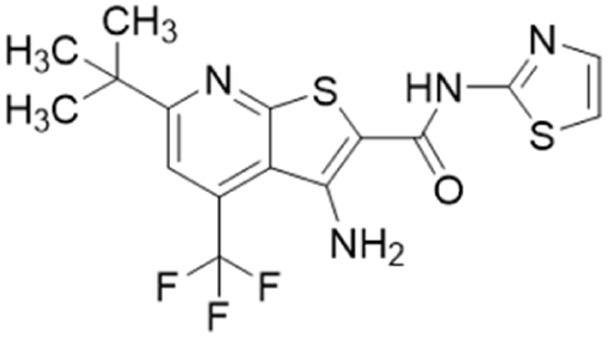	*K*_i_ = 2 μM	Kami Reddy *et al.*	2019	13
8a^a^ (46)	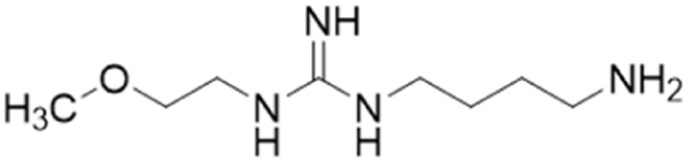	*K*_i_ = 18 μM	Lunk *et al.*	2020	28

a Published identification number.

In this study, we review the literature of known inhibitors of *h*DDAH1 over the past two decades (patents not included) considering their structure activity relationships (SARs) and X-ray crystal structures. Furthermore, we discuss current challenges in designing and developing novel inhibitors for DDAH1 and suggest future directions.

### Butanoic acids as DDAH1 inhibitors

In one of the earliest studies, MacAllister *et al.*^16^ identified 1 (4124W) as a weak inhibitor of rat and human DDAH (IC_50_ = 416, 250 μM) respectively. This inhibitor is a short-chained analogue of L-NMMA and the first known reversible inhibitor of mammalian DDAH. This compound exhibited endothelium-dependent vasoconstriction in mouse models (evidence of indirectly inhibiting NO production),^17^ and was chosen as the lead compound for reversible DDAH inhibitor design by Rossiter *et al.*^18^

**Fig. 2:**
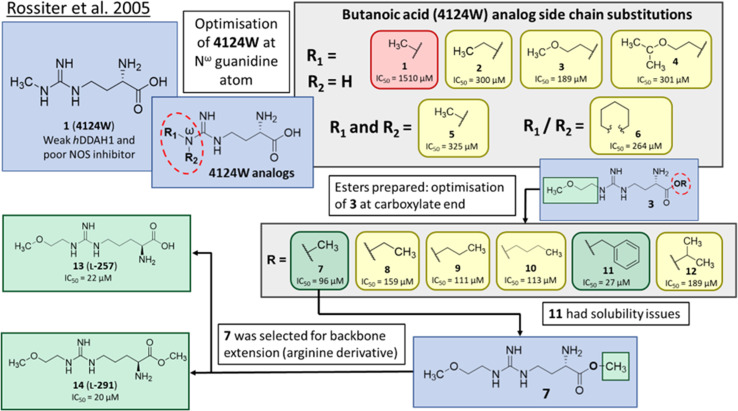
Development of DDAH1 inhibitor L-257 (13)^18^ from 4124W (1).^16^ Optimisation at N^ω^-guanidine and carboxylate ends. N^ω^ side chain and carboxy substitutions shown in green (*K*_i_ < 100 μm); yellow (*K*_i_ = 100–1000 μm) and red (*K*_i_ > 1000 μm).

Optimisation of 1 was initiated by modifying the N^ω^ guanidine atom, Fig. 2. The short carbon chain length of 1 was initially maintained by changing the substituents at the guanidine N^ω^ atom. Under these conditions, 1 did not show promising activity (1, IC_50_ = 1510 μM). Increasing the C chain from methyl (1) to ethyl (2) further improved activity (2, IC_50_ = 300 μM). Greater improvement was observed introducing an O atom, forming 2-methoxyethyl (3, IC_50_ = 189 μM). However, activity decreased when introducing isopropoxyethyl functional group (4, IC_50_ = 301 μM). Dual methyl groups at this position gave similar potency (5, IC_50_ = 325 μM) but also inhibited eNOS and was abandoned. Interestingly, introducing a sterically larger piperidinyl group marginally improved activity (6, IC_50_ = 264 μM). The most potent analog, 2-methoxyethyl, was retained for further studies (3, IC_50_ = 189 μM). Ester modifications at the carboxyl end of 3 were further optimised.

The short methyl ester modification further improved ligand activity (7, IC_50_ = 96 μM) compared to 3 (IC_50_ = 189 μM). Linear ethyl ester modification moderately improved ligand activity (8, IC_50_ = 159 μM). Extension of the aliphatic C chain to propyl ester (9, IC_50_ = 111 μM) and butyl ester (10, IC_50_ = 113 μM) improved DDAH inhibition. Interestingly, a larger aliphatic benzyl group gave the efficient ligand activity (11, IC_50_ = 27 μM) but was abandoned by the authors due to poor solubility. Isopropyl ester (12, IC_50_ = 189 μM) did not alter ligand activity compared to the standard carboxy acid group (3, IC_50_ = 189 μM). As a result, developing a methyl substitution (7) was one of the obvious choices for the authors.

### Arginine derivatives as DDAH1 inhibitors

Despite possible NOS inhibition, Rossiter *et al.*^18^ explored arginine derivatives (l-arginine is a substrate of NOS) for developing DDAH1 inhibitors.^19,20^ Compound 7 was further developed to arginine derivatives by extending the carbon backbone which improved DDAH1 activity significantly. Carboxylic acid motif 13 (IC_50_ = 22 μM, Fig. 2) gave similar potency to the methyl ester (14, IC_50_ = 20 μM) and was favoured, as 14 is anticipated to be converted to 13 by liver carboxylesterases.^21^ Additionally, the removal of carboxy or 2-amino groups, R-enantiomers of the most active compounds, and cyclic analogues did not improve DDAH1 inhibition. Overall, 13 was identified as a potent and selective DDAH1 inhibitor. The methyl ester analogue 14 exhibited elevated plasma ADMA concentrations indicating DDAH1 inhibition in mouse models.

### Arginine side chain and carboxylate bioisosteres of L-257

**Fig. 3:**
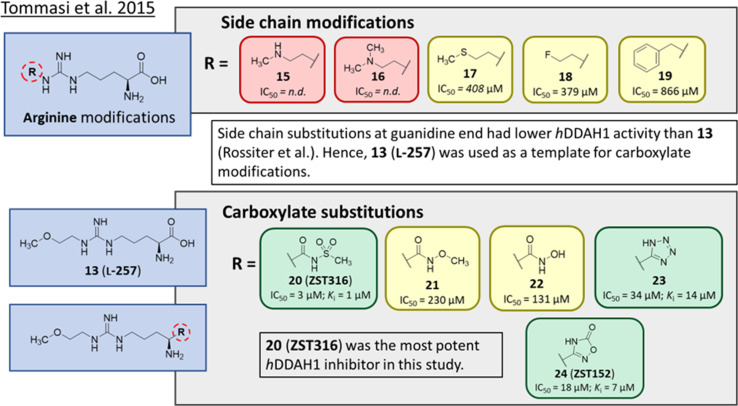
Development of *h*DDAH1 inhibitor ZST316 (20)^22^ from L-257 (13).^18^ R group substitutions shown in green (*K*_i_ and IC_50_ < 100 μm); yellow (IC_50_ = 100–1000 μm) and red (IC_50_ = n.d.). n.d. = not determined when inhibition <50% at 1 mm.

Tommasi *et al.*^22^ developed a set of inhibitors based on 13, due to the selectivity of this scaffold for DDAH over NOS and arginase.^18,23^ Two classes of inhibitors were explored: 13 side chain derivatives (substitution at the 2-methoxyethyl end) and 13 carboxylate bioisosteres, Fig. 3. 1,2,3-Triazoles with different chemical structures to 13 were also explored, however, these compounds displayed poor solubility and DDAH1 inhibition.

Side chain derivatives with diverse substituents were investigated with alterations at the 2-methoxyethyl end of 13. Replacement of an oxygen for a nitrogen at the 2-methoxyethyl end gave 15 (IC_50_ = n.d.; 29% at 1 mM) with weaker affinity for *h*DDAH1. A modified tertiary amine group (16, IC_50_ = n.d.; 14% at 1 mM) also gave poor inhibition. Hydrophobic interactions were explored using methylthio group (17, IC_50_ = 408 μM), with moderate *h*DDAH1 inhibition. Altered electronegativity using 2-fluoroethyl group (18, IC_50_ = 379 μM) was the best of this series but still less potent than 13 (IC_50_ = 22 μM, Fig. 2). A benzyl group side chain was investigated, but did not significantly inhibit *h*DDAH1 (19, IC_50_ = 866 μM).

All carboxylate bioisosteres inhibited the enzyme at 1 mM concentrations (79–98%). The most potent of this series was acylsulfonamide (20, ZST316, IC_50_ = 3 μM; *K*_i_ = 1 μM). There was a decrease in inhibition of *h*DDAH1 when the acidity of the bioisosteric replacement was less than the carboxylate group of 13, O-methylhydroxamic group (21, IC_50_ = 230 μM) and the hydroxamic group (22, IC_50_ = 131 μM). However, introducing bioisosteres with multiple heteroatoms, such as tetrazole (23, IC_50_ = 34 μM; *K*_i_ = 14 μM) and oxadiazolone (24, ZST152, IC_50_ = 18 μM; *K*_i_ = 7 μM) significantly restored *h*DDAH1 inhibition. According to the authors, positively charged residues at the *h*DDAH1 active site may facilitate the binding of inhibitors with greater acidity near the carboxylate end of the (13, L-257) scaffold.

**Fig. 4:**
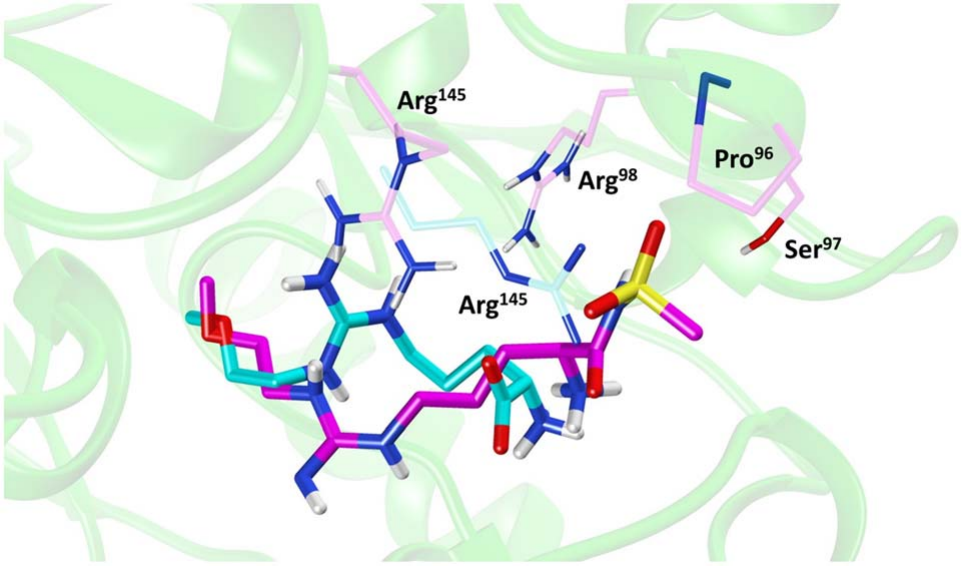
Binding mode of ZST316 from molecular dynamics simulation^22^ overlayed on the X-ray crystal structure of L-257 bound to *h*DDAH1 (pdb 2jaj). Image created using UCSF Chimera.

Molecular dynamics (MD) simulations of 20 revealed a novel binding mode within the *h*DDAH1 binding site, Fig. 4. It was shown that this inhibitor induces a change in the rotamer position of Arg145 (protein numbering Met = 1) by exploiting a new binding pocket. Aided by main-chain atoms from helix 4 residues, a sulfonyl O atom forms four H-bonds with the main chain NH of Pro96 and Ser97, with the side chain OH of Ser97 and with the side chain NH of Arg98. The second O atom of the sulfonyl group H-bonds with the side chain NH of Arg145. The authors proposed that these novel interactions of 20 (not noted in 13) within DDAH1 are likely responsible for increased DDAH1 inhibition.

### Ornithine and arginine derivatives as DDAH1 inhibitors

**Fig. 5:**
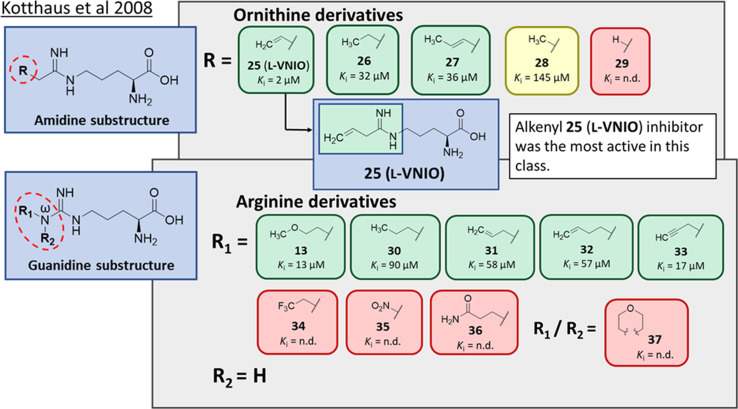
Development of *h*DDAH1 inhibitors using amidine and guanidine substructures.^26^ L-257 (13) activity as *K*_i_ value. R group substitutions shown in green (*K*_i_ < 100 μm); yellow (*K*_i_ = 100–1000 μm) and red (*K*_i_ = n.d.). n.d. = not determined when inhibition <50% at 1 mm.

Ornithine derivatives with an amidine functional group (L-VNIO and associated analogs) were previously identified as potent NOS inhibitors.^24,25^ Amidines are bioisosteres of guanidine and expected to have similar *h*DDAH1 potency as derivatives of l-arginine. Kotthaus *et al.*^26^ integrated similar analogs by substituting amidine side chains, evaluating linear aliphatic and alkenyl side chain groups. These derivatives probed the effect of chain length and double bonds on *h*DDAH1 inhibition, Fig. 5.

Ornithine derivatives with an amidine substructure and simple aliphatic substitutions were evaluated for DDAH1 inhibition. Ethylene was found to be the most potent from this series (25, L-VNIO, *K*_i_ = 2 μM). Removing the electron density and changing the geometry of the terminal C atom from a planar sp^2^ hybridised π-bond (25) to a tetrahedral sp^3^ hybridised ethyl group (26, *K*_i_ = 32 μM) did not show significant improvement in DDAH1 inhibition. Also, extending the C chain to form the *trans*-alkenyl analog (27, *K*_i_ = 36 μM) did not improve inhibitory activity. Smaller substitutions such as methyl (28, *K*_i_ = 145 μM) and hydrogen (29, *K*_i_ = n.d.) gave unfavourable *h*DDAH1 inhibition.

Arginine derivatives with a guanidine substructure (30–37, Fig. 5) were developed to improve *h*DDAH1 inhibition. These molecules were compared to 13 (*K*_i_ = 13 μM, Fig. 5) as they all contain guanidine and were expected to bind similarly to the DDAH1 active site. Removing the O atom from 2-methoxythyl end decreased ligand activity (30, *K*_i_ = 90 μM). However, π-electron density at the terminal position (31, *K*_i_ = 58 μM) partly restored ligand activity. Extending the C chain of 31 by 1C atom did not improve DDAH1 inhibitory effects (32, *K*_i_ = 57 μM). Further enhancement was observed by the alkyne derivative (33, *K*_i_ = 17 μM), suggesting stronger electronic effects (sp hybridisation) at this position favouring *h*DDAH1 inhibition. The least active compounds in the guanidine series had side chain substitutions with electron withdrawing properties, *e.g.*, trifluoromethyl (34, *K*_i_ = n.d.) and nitro (35, *K*_i_ = n.d.) groups. Analogs with slightly larger steric bulk, such as N^ω^-(2-carbamoylethyl)-l-arginine (36, *K*_i_ = n.d.) and the cyclic N^ω^-morpholinyl-l-arginine (37, *K*_i_ = n.d.) were also less effective than the alkenyl derivatives. Importantly, the electron donating 2-methoxyethyl group (13, *K*_i_ = 13 μM) was more active than the electron withdrawing groups (34 and 35, *K*_i_ = n.d.) or those that are sterically larger (36 and 37, *K*_i_ = n.d.). Additionally, it appears the *h*DDAH1 active site is sensitive to the size of functional groups at the N^ω^ atom position, as relatively sterically large substitutions at this position appear to hinder *h*DDAH1 inhibition. Additionally, the rigidity of the functional group at the N^ω^ position is undesirable as shown in ref. 37. As a result, flexibility of substitution may be a prerequisite for optimal binding.

Testing of the ornithine and arginine derivatives against NOS showed potent inhibition towards all NOS isoforms (25–29 were previously known inhibitors of NOS). However, 13, 27, 32, 34, 36 and 37 were less effective NOS inhibitors. The inhibition of *h*DDAH1 by 27 and 32 was particularly interesting since there was selectivity for *h*DDAH1 over NOS.

The authors suggested that elongation of the alkenyl side chain may improve selectivity towards DDAH1 over NOS. This hypothesis is supported by the work of Rossiter *et al.*,^18^ who demonstrated that larger substitutions such as 2-isopropoxyethyl (4, IC_50_ = 301 μM, Fig. 2) may bind to the DDAH active site. This is further supported by the fact that molecules larger than N^ω^ propyl cannot fit inside the active site of NOS,^27^ where N^5^-(1-iminohexyl)-l-ornithine (Butyl-L-NIO) inhibits NOS weakly (>1.5 mM).^25^

The first *h*DDAH1 inhibitor with amidine substructure having excellent DDAH1 activity was 25 (L-VNIO, *K*_i_ = 2 μM, Fig. 5). However, this compound was not selective over NOS when compared to 13 (L-257, *K*_i_ = 13 μM, Fig. 5).

### Advancement of ornithine and arginine DDAH1 inhibitors

Rossiter *et al.*^18^ previously developed 13 (L-257, Fig. 2 and 5), a selective inhibitor towards *h*DDAH1 over NOS and arginase. Likewise, Kotthaus *et al.*^26^ aimed to develop more potent and selective *h*DDAH1 inhibitors using ornithine and arginine side-chain substitutions, Fig. 5. They identified 25 (L-VNIO, *K*_i_ = 2 μM) that was found to be more potent than 13 (*K*_i_ = 13 μM) but less selective towards *h*DDAH1 over NOS and arginase,^23^ prompting further investigation. Furthermore, it was proposed that the 2-methoxyethyl substituent of 13 is not favourable for binding to the NOS active site (possibly a consequence of inappropriate steric and/or electrostatic interactions) hence more selective for DDAH1.^23,26^ Therefore, following from Kotthaus study, Lunk *et al.*^28^ investigated a diverse range of methoxy and alkenyl substitutions by shortening and extending the C side chain, leading to improved potency and selectivity towards *h*DDAH1. Major advancements from this study include the identification of a novel selective *h*DDAH1 inhibitor with a novel binding mode. In addition, it provided conclusive evidence that the α-carboxy group is dispensable for potent *h*DDAH1 inhibition for arginine-based inhibitors.

**Fig. 6:**
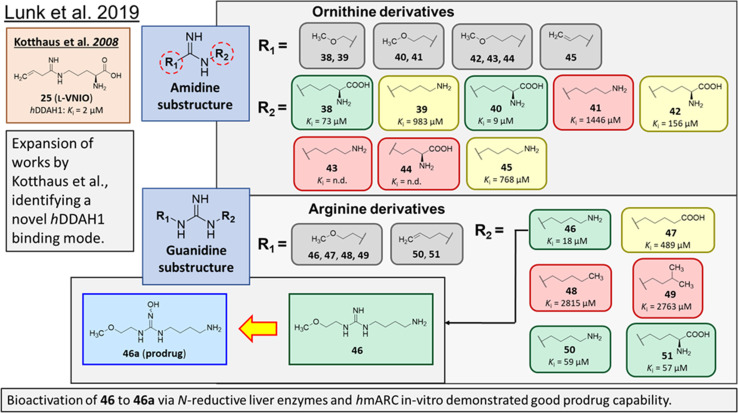
Development of *h*DDAH1 inhibitor 46 from guanidine substructure.^28^ R group amidine and guanidine substitutions are shown in green (*K*_i_ < 100 μm); yellow (*K*_i_ = 100–1000 μm) and red (*K*_i_ > 1000 μm or n.d.). n.d. = not determined when inhibition <20% at 1 mm.

Ornithine and arginine derivatives having amidine and guanidine substructures respectively are shown in Fig. 6. Ornithine analogs with a 2-methoxy substituted side chain (38 and 39) was most potent when both carboxy and amine groups were present (38, *K*_i_ = 73 μM) and less potent when removing the carboxy but retaining the amine group (39, *K*_i_ = 983 μM). Simarily, analogs with a 3-methoxy side chain (40 and 41) were more potent with the carboxy group (40, *K*_i_ = 9 μM) than without (41, *K*_i_ = 1446 μM). Although 40 gave similar potency to 13 (*K*_i_ = 13 μM, Fig. 5), was less selective over NOS and arginase. Increasing the side chain length to 4-methoxy restored *h*DDAH1 inhibition somewhat (42, *K*_i_ = 156 μM) but activity was again lost without the carboxy group (43, *K*_i_ = n.d.). Interestingly, poor ligand activity was observed when shortening the main C chain (44, *K*_i_ = n.d., supported by previous results of *nor*-arginine and *nor*-ornithine butanoic acid analogs^18^) or when a propene side chain was introduced (45, *K*_i_ = 768 μM). In summary, ornithine analogs without the α-carboxy group were less potent than those with both the α-carboxy and amine groups.

Arginine derivatives incorporating guanidine were compared to 13 (*K*_i_ = 13 μM, Fig. 5).^26^ Interestingly, the most potent analog from this series incorporated the 2-methoxyethyl side chain with the removal of the α-carboxy group (46, *K*_i_ = 18 μM) which gave comparable potency and selectivity to 13 over NOS and arginase. Conversely, absence of the α-amine group but presence of the α-carboxy group decreased inhibitory potency (47, *K*_i_ = 489 μM). Pentyl substitution (48, *K*_i_ = 2815 μM) or isopentyl (49, *K*_i_ = 2763 μM) was not favourable towards *h*DDAH1 inhibition. However, butyl amine (50, *K*_i_ = 59 μM) and amino pentanoic acid (51, *K*_i_ = 57 μM) groups restored ligand activity.^28^

Notably, arginine analog 46 (*K*_i_ = 18 μM) gave comparable potency and selectivity to L-257 (13, *K*_i_ = 13 μM, Fig. 5) over NOS and arginase. This suggests the electronic effects of 2-methoxyethyl guanidine group contributes significantly to *h*DDAH1 binding. Importantly, the equivalent amidine analog (41, *K*_i_ = 1446 μM) was not as effective, indicating that the positive charge induced by electron delocalisation of the guanidine group is essential for *h*DDAH1 inhibition. Interestingly, the decarboxylated guanidines (46 and 50) were more selective towards *h*DDAH1 over NOS and arginase than their carboxylate partners. This suggests the α-carboxy group is dispensable for potent and selective *h*DDAH1 inhibition using arginine (guanidine) derivatives. Overall, removal of the α-carboxy group was tolerated by guanidine (arginine) but not amidine (ornithine) functional groups.

**Fig. 7:**
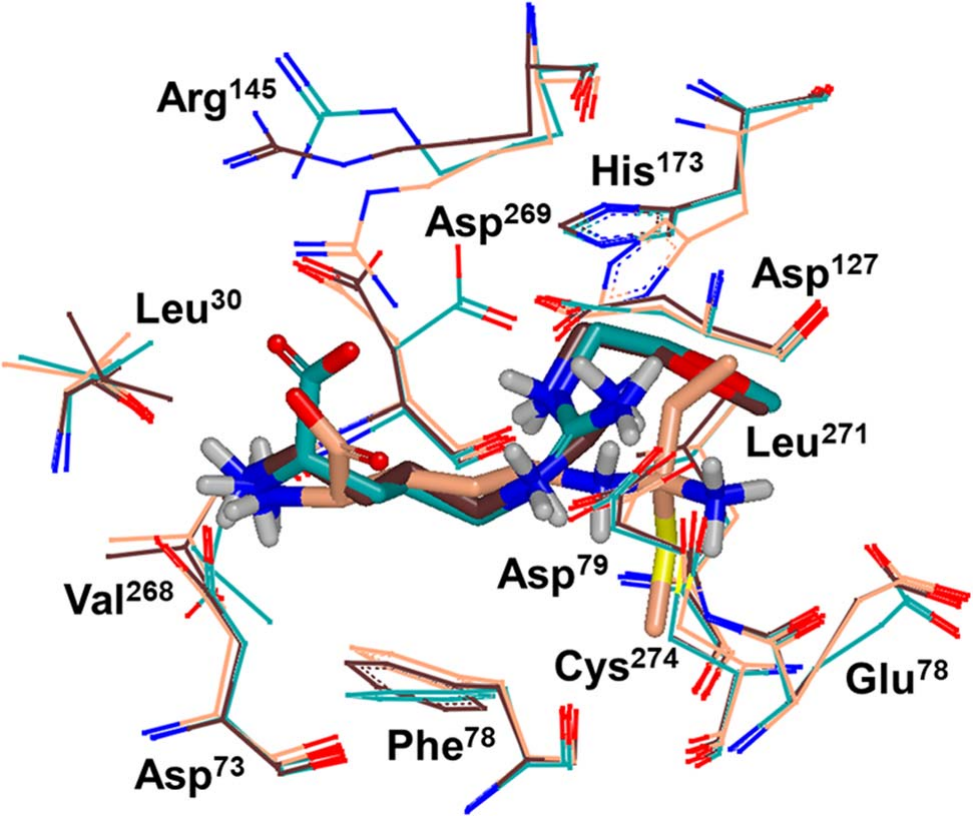
X-ray crystal structure overlay of *h*DDAH1 bound to 13 (L-257) (cyan, pdb 2jaj), 46 (brown, pdb 6szp) and 59 (L-IPO) (tan, pdb 3i4a). H, N, O and S atoms are shown in white, blue, red and yellow respectively. Residues numbered, Met = 1. Image created using OpenEye VIDA.

Binding modes of 13 (pdb 2jaj) and inhibitor 46 (pdb 6szp) bound *h*DDAH1 were defined using X-ray crystallography. Superposition of 13 and 46 show almost identical binding modes, Fig. 7. Comparison of these inhibitors bound to *h*DDAH1 (ref. 28) all show the α-amino group positioned tightly by H-bonds, interacting with the side chain of Asp73 and backbone carbonyl interactions of Leu30 and Val268. This highlights the importance of the α-amine group likely due to the optimal geometry of all H-bond partners. Noticing the similar alignment of the butyl chains of 13 and 46, orientation appears to originate from their guanidine groups. Crystal structures of 13 and 46 show the outward pointing guanidine NH interacts with Asp79 and the aminobutyl substituted guanidino-NH with Asp79. A second set of interactions bind the methoxyethyl-substituted distal guanidino-NH on the opposite side in place *via* side chain and backbone carbonyl oxygens of Asp269. Therefore, Asp79 and Asp269 residues form 2 clamps securing the guanidine group in place. These clamps influence the positioning of the 2-methoxyethyl and 4-aminobutyl groups in the active site. This strong interaction of guanidine and the two Asp guanidine clamps induce a rotation of His173 out of its apo (native) position, causing an outward twist of Arg145. This is opposite to the N^5^-(1-iminopentyl)-l-ornithine (L-IPeO)^29^ and l-citrulline^30^ binding modes (pdb 3p8p and 2jai respectively), as the respective amidino or urea groups cannot interact with Asp79/269 in the same way. The difference is the greater flexibility of the pentyl (L-IPeO) chain and the amino acid side chain (L-IPeO, l-citrulline), compared with 13 and 46. The authors suggest the higher basicity of guanidine also induces stronger binding compared to amidine or urea. Lacking the guanidine preorientation interactions of an ornithine (amidine) based inhibitor, appears to have a higher degree of freedom enabling interaction of the α-carboxy group with Arg145. This interaction is responsible for potent binding and without the COOH group, results in loss of activity towards *h*DDAH1. Interestingly, the gauche conformation of the butyl chain (46) is tolerated due to strong binding *via* the guanidino and α-amino groups. In contrast, amidine groups adopted anti conformations suggesting weaker interactions. (This guanidine binding of the butyl chain appears to make the Arg145 interaction dispensable, but the α-amine group is necessary for potent binding).

Drug-like qualities of 46 were considered for optimal absorption *via* passive diffusion. As 13 has zwitterionic character, absorption was expected to be mediated by active uptake of amino acid transporters. However, this is feasible for the primary amine of 46, but its guanidine component would be fully charged under physiological conditions preventing membrane transport by passive diffusion. Therefore, a prodrug *N*-hydroxyguanidine 46a, formed by hydroxylating the central N^ώ^ atom of guanidine of 46 was further investigated.

As demonstrated in their previous works, *N*-hydroxylated guanidines are effectively bioactivated by the mARC (mitochondrial amidoxime-reducing component) containing N-reductive enzyme system *in vitro*, with evidence of bioactivation using this system *in vivo*.^31^ Therefore, 46a is expected to be reduced to 46 efficiently in the liver *via in vivo* bioactivation.

vvInvestigations of distinct subcellular liver fractions showed highest reduction rates of 46a in outer mitochondrial membrane vesicles (OMV) followed by mitochondria and liver homogenate. Additionally, incubations with reconstituted heterologously expressed human mARC-1 and mARC-2 isoenzymes confirmed that 46a is a particularly good substrate of the N-reductive enzyme system. Based on these results, there is high probability of acceptable bioavailability of 46a. One concern was the chemical stability of *N*-hydroxyguanidines, however, 46a was stable at different pH's over 24 h at 37 °C. Both the guanidine and prodrug 46a showed excellent profile with respect to cell toxicity/viability that will be useful as a pharmacological toolset.

### Irreversible DDAH1 inhibitors

**Fig. 8:**
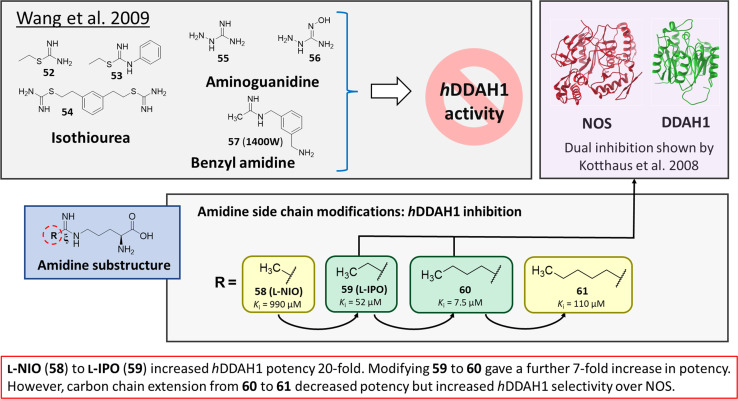
Rational drug design by Wang *et al.*^32^ R group substitutions shown in green (*K*_i_ < 100 μm) and yellow (*K*_i_ = 100–1000 μm).

Wang *et al.*^32^ developed dual NOS/DDAH1 inhibitors from ornithine derivatives, expected to affect NO biosynthesis greater than single targeted compounds. A set of previously known NOS inhibitors were selected for study, (52–57, Fig. 8). *S*-Alkyl isothioureas (52–54) and aminoguanidines (55 and 56) were initially assayed but were not respective substrates or inhibitors of *h*DDAH1 so were not pursued further by the authors. Benzyl amidine based 1400W (57) (selective iNOS inhibitor) did not inhibit *h*DDAH1 at concentrations <1 mM. However, alkyl amidine inhibited weakly (58, L-NIO, *K*_i_ = 990 μM), showing potential for dual NOS/DDAH1 inhibitors. Wang *et al.*^32^ proposed the α-amino and carboxylate groups may contribute to binding in the active sites of NOS and DDAH1, which was validated by Lunk *et al.*^28^ Alternatively, an expanded set of alkyl-substituted ornithine derivatives were synthesised and assayed towards DDAH1 and compared to NOS inhibition.

Extending the carbon chain of L-NIO (58, *K*_i_ = 990 μM) by 1 methyl group to N^5^-(1-iminopropyl)-l-ornithine (L-IPO, 59, *K*_i_ = 52 μM, Fig. 8) increased *h*DDAH1 potency by 20-fold. Extending 59 by an additional 2 methyl groups (60, *K*_i_ = 7.5 μM) increased potency a further 7-fold. Branching the alkyl chain did not improve potency. The extension of the carbon chain decreased *h*DDAH1 ligand activity (61, *K*_i_ = 110 μM) suggesting space at the active site is limited, however, selectivity over NOS is increased. The trend of side chain substitutions is similar for both *h*DDAH1 and *h*NOS. For example, as the number of carbon atoms is increased, the affinity towards each enzyme decreases albeit with different magnitudes.

X-ray crystal structure^32^ shows continuous electron density between the active site Cys274 and the amidino carbon (C^ζ^) of 59 (Fig. 7), indicating covalent bond formation suggesting a tetrahedral sp^3^ complex. Notably, solution studies suggest this compound exhibits a reversible covalent competitive inhibition. The orientation of the (R)-inhibitor complex directs the orientation of the terminal side chain methyl group in the same pocket as the substrate's leaving group (ADMA, N^ω^-CH_3_). This may indicate hydrophobic interactions with Leu271. The amidine N^ω^ atom also appears to make a H-bond or ionic interaction with the carboxylate side chain O atom of Glu78. Both charged and hydrophobic interactions of the amidine group contribute to the affinity of 59 towards *h*DDAH1. This was demonstrated using site-directed mutagenesis studies of amino acid residues within the DDAH1 binding site.

A follow-up study by Lluis *et al.*,^29^ showed that compound 60 (*K*_i_ = 7.5 μM, Fig. 8) showed a similar binding mode to 59 (*K*_i_ = 52 μM). The X-ray crystal structure showed a covalent bond between Cys274 and C^ζ^ atom of 60 with tetrahedral geometry. Additionally, the carboxylate and amino groups of the ligand make non-covalent interactions with the side chain carboxylate of Asp73 and carbonyl O atoms of Leu30/Val268. The N^ε^/N^ω^ amidine atoms (either side of C^ζ^) of 60 form H-bonds with side chain O atoms of Asp79. The alkyl substituent of 60 additionally forms hydrophobic contacts in the active site pocket made up of Leu271, His173 and Gly129. The alkyl substituent of 60 is also packed against His173 more orderly than 59, likely adding additional van der Waals forces increasing potency. Overall, Wang *et al.*^32^ found that NOS inhibition requires amidines with alkyl substituents between 2 and 5 carbons in length (58, 59, 60) but DDAH1 inhibition requires between 3 and 6 carbons in length (59, 60, 61).

### Structure based design and discovery of DDAH1 inhibitors

**Fig. 9:**
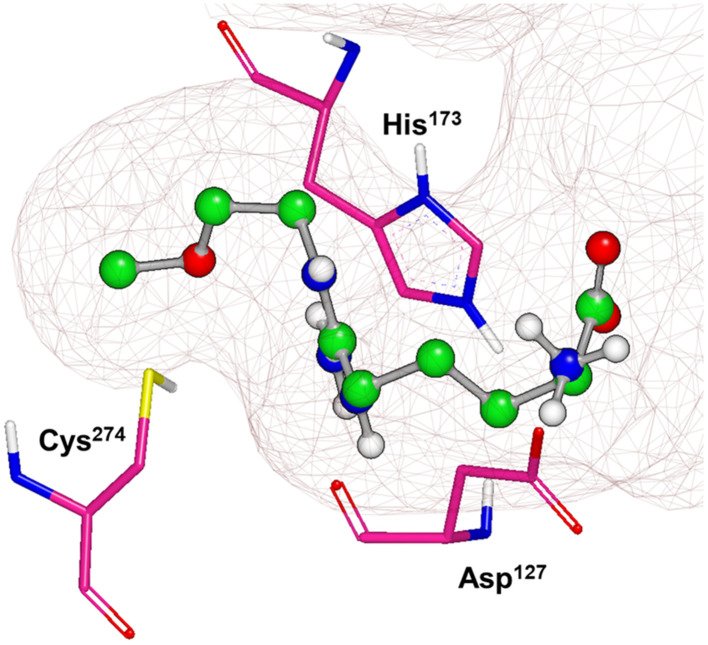
X-ray crystal structure of *h*DDAH1 (pdb 2jaj), with key catalytic residues displayed. Image created using OpenEye VIDA.

X-ray crystal structures of bacterial (*Pseudomonas aeruginosa*)^30^ and mammalian (bovine^33^ and human^34^) DDAH1 have been resolved. The DDAH1 enzyme is similar to the arginine/glycine amidino transferases,^30^ consisting of a 5 stranded α/β propeller-like fold where the substrate binds in the centre of a “barrel” or cavity at the protein centre and undergoes catalysis (Fig. 9). The barrel at one end is closed by a lid or “flap” made from a looped polypeptide chain. The DDAH1 active site lies in a negatively charged cleft at the centre of the barrel of its propellor-like fold.

The first human X-ray crystal structures of DDAH1 were resolved in the presence of 13 (pdb 2jaj) and citrulline (pdb 2jai),^34^ where residues Cys274, His173 and Asp127 (protein numbering Met = 1) form the catalytic triad (associated with substrate metabolism). Key H-bonding interactions were shown between guanidine atoms (N^ω^ and N^δ^) and Asp79.^34^ The aliphatic portion of the ligand (13) backbone packs against Phe76. The amino N atoms form H-bonds with Asp73 and to the main chain O atoms of Leu30 (part of the “lid” region) and Val268. Notably, different orientations of His173 and Arg145 side chains were observed when *h*DDAH1 is bound to l-citrulline (pdb 2jai),^34^ suggesting ligand induced conformational differences in the DDAH1 binding site.

A comparison of an irreversible covalent inhibitor (59, pdb 3i4a, Wang *et al.*^32^) to two reversible inhibitors, 13 and 46, revealed similarities and differences in binding mode and amino acid side chain flexibility, (Fig. 7). The backbone of 13 and 46 has a similar mode of ligand binding, whereas 59 has a distinctly different position. When compared with both reversible inhibitors (13 and 46), His173 and Leu30 align differently with 59 at the DDAH1 binding site. It is not surprising that Cys274 adopts a different orientation when covalently bound to 59 than the reversible inhibitors. However, residues from 59 (Asp73 and Glu78) show similar conformations as those of 46 bound *h*DDAH1, suggesting removal of the carboxy group may activate the binding site simarily to 59. Alternatively, when 13 is bound to *h*DDAH1, Leu30, Asp73 and Glu78 are positioned slightly differently than 46 and 59.

**Fig. 10:**
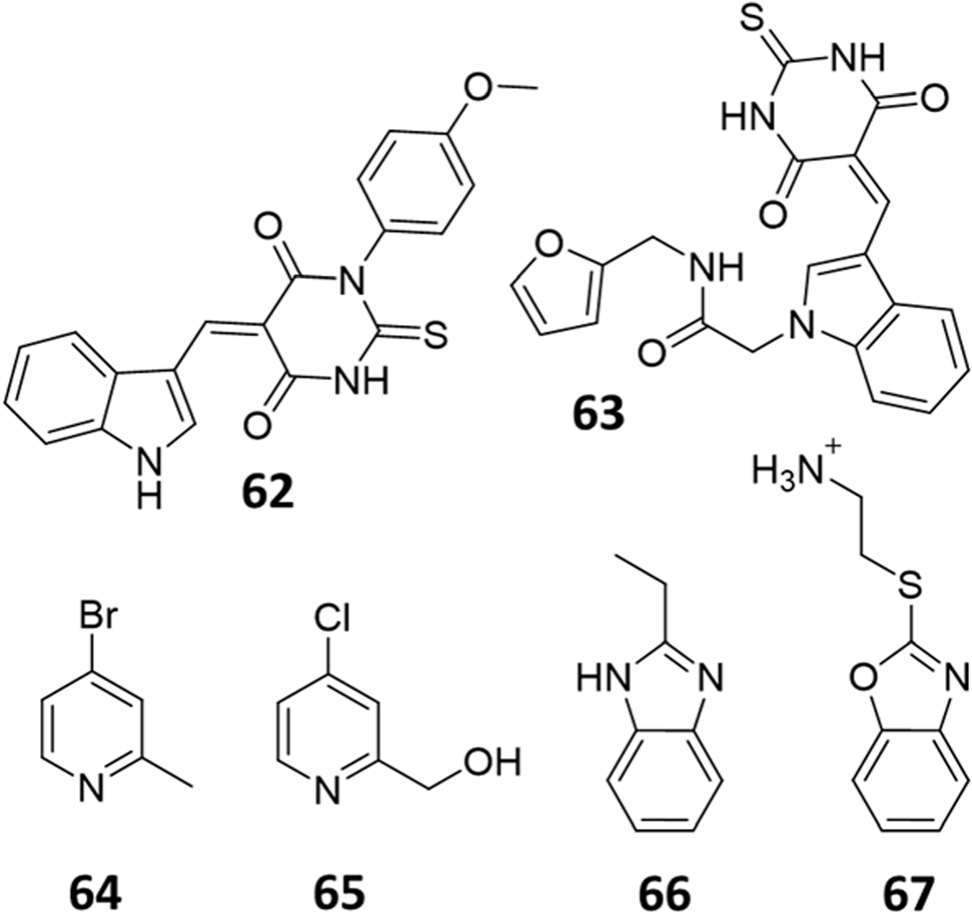
*Pa*DDAH1 inhibitors 62 and 63, identified by Hartzoulakis *et al.*^36^*via* virtual screening and hit analysis. *h*DDAH1 inhibitors 64, 65, 66 and 67, identified by Linsky *et al.*^35^*via* fragment based high-throughput screening (HTS).

Only two studies have reported the utilisation of X-ray crystal structures in the discovery of diverse and novel DDAH1 inhibitors.^35,36^ However, none of these studies have optimised the identified compounds (non-arginine scaffolds) into potent DDAH1 inhibitors. Hartzoulakis *et al.*^36^ identified potent *Pa*DDAH1 inhibitors with different chemical structures than typical arginine-based inhibitors. A database of 308 000 commercially available compounds were filtered to remove non-drug-like compounds, reducing the number to ∼260 k. Reciprocal nearest neighbour (RNN) packing algorithm was then used to generate a second subset of active compounds, reducing the total number to 35 000 structures. Fragment based docking was conducted using FlexX. The top 200 ranked molecules were assessed for binding at the *Pa*DDAH active site (at least 1 H-bond between protein and ligand). Out of the top scored compounds, 90 were purchased and screened using colorimetric assay. Two potent actives (62, 63) were identified from this study (Fig. 10). However, the most potent inhibitor 62 from this study did not inhibit *h*DDAH and several compounds showed poor solubility.^26,36^ Thus, this study highlights that *Pa*DDAH1 may not be an optimal screening system for developing human DDAH1 inhibitors due to differences in the ligand binding site.

In another study, Linsky *et al.*^35^ performed a high through-put screening (HTS) of 4000 molecules against *Pa*DDAH and *h*DDAH1 isoforms to find a DDAH inhibitor with diverse structure. This HTS utilised an alternative DDAH substrate, *S*-methyl-l-thiocitrulline, to produce an alternative product, methanethiol. This thiol is detected by chromogenic or fluorogenic reagent and this increase in signal is compared against control wells to observe any inhibition by library compounds. Both isoforms (*Pa*DDAH and *h*DDAH) were assayed with the intention of increasing the probability of finding DDAH inhibitors. From a 4000-member fragment library, 101 unique molecules were identified with potential inhibition for *Pa*DDAH and *h*DDAH1. These “hits” were manually categorized by structure and a representative from each was repurchased for validation tests, resulting in a total of 107 compounds that progressed to further study. A series of validation tests were designed to eliminate false positives, where 68% of the initial primary hits were identified as false positives due to assay interference. Two additional validation tests were used to eliminate false positives to the 31 hits identified in the second assay. Four previously unknown inhibitors (64–67) were identified from this study, Fig. 10. Two of these small molecule inhibitors (64, 65) have a 4-halopyridine scaffold that function as covalent quiescent affinity labels and two are benzimidazole-like inhibitors that reversibly and competitively inhibit human DDAH1. Ebselen was also identified as a potent *h*DDAH1 inhibitor from this study, however, this compound binds to multiple targets^37–39^ therefore complicating its use in the clinical setting. A high number of false positives noted in this study highlights the potential challenges in screening new DDAH1 inhibitors.

### Current challenges in the development of DDAH1 inhibitors

**Fig. 11:**
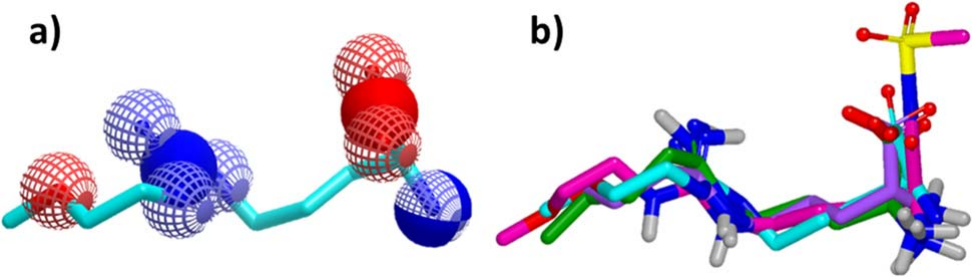
(a) Hydrogen bond acceptor (red) and donor (blue) atoms of L-257 (13). (b) Overlay of *h*DDAH1 inhibitors 13 (L-257, cyan), 20 (ZST316, magenta), 26 (purple) and 60 (green). H, N, O and S atoms are shown in white, blue, red and yellow respectively. Images created using OpenEye vROCS^50^ and VIDA.^51^

Since its first discovery of DDAH1 inhibitors about three decades ago there has been slow progress in the development of effective inhibitors. For instance, (i) the current DDAH1 inhibitors have low potency in the micromolar (μM) range; (ii) only a few inhibitors are selective for DDAH1; (iii) almost all DDAH1 inhibitors are based on the arginine scaffold; (iv) the pharmacokinetic characteristics of existing inhibitors have not been thoroughly tested and (v) not a single DDAH1 inhibitor has progressed to human trials.^9^ The main reason for this is that compounds other than arginine/ornithine have not been researched thoroughly for DDAH1 inhibition. It is evident from a comparison of pharmacophore features and the chemical backbone of existing potent *h*DDAH1 inhibitors 13, 20, 26, and 60 (Fig. 11) that there is a lack of chemical diversity among these compounds.

DDAH1 inhibitors that mimic the natural substrate (ADMA) are attractive because they are expected to bind to the active site and transported throughout the mammalian body, simarily to ADMA. Moreover, majority of the arginine based analogues are relatively easy to synthesise with decent yields. However, major limitations of known DDAH1 inhibitors displaying non-specific interactions with NOS and arginase.^26,28,32^ Also for most of the DDAH1 inhibitors (both arginine and non-arginine-like) limited information of their pharmacokinetic profiles are available with only a few studies performed in animal models. Pharmacokinetic data is available for L-257 (13), ZST316 (20) and ZST152 (24)^40,41^ and animal studies detailing the effects of DDAH1 inhibition of L-257 (ref. 34) and DD1E5.^13^

Previously, Rossiter *et al.*^18^ tested the selectivity of 13 and 14 (L-257 and L-291 respectively, Fig. 2) towards DDAH1 over other NOS isoforms and were found to be selective for DDAH1. 14 was further characterised for the inhibition of DDAH *in vivo*.^34^ After administering the inhibitor in mice, plasma ADMA concentrations increased to concentrations that are commonly observed in patients with multiple cardiovascular risk factors.

A recent study conducted by our research group^41^ assessed the pharmacokinetics of 20 and 24 (ZST316 and ZST152 respectively, Fig. 3), inhibitors of *h*DDAH1. Intravenous administration of 20 (1.63 h, 60 mg kg^−1^) displayed a slightly longer half-life compared to 24 (0.86 h at 60 mg kg^−1^) in mice. However, oral bioavailability was greatest for the lipophilic moiety (24, *F* = 33.3%) than sulfonamide analogue (20, *F* = 4.7%). Sulfonamides are commonly hydrolysed by hepatic carboxylesterase but they may be useful as a prodrug.^42^ Intraperitoneal administration further assessed the pharmacokinetic parameters of 20. Improved characteristics were observed at 30 mg kg^−1^ dosage with a half-life of 8 and 6 h, bioavailability of 59–67% and urinary excretion of 54–56% by chronic and acute treatment, respectively. Bioavailability of 20 indicates >50% absorption and excretion concentrations, suggesting the drug is not over metabolised. However, further studies are required to determine whether these compounds display enough drug-like properties for use in humans.

Enzyme inhibitors are often designed with a single target in mind to minimise off-target effects. However, no definitive conclusions have been available on whether dual DDAH1/NOS inhibition could significantly reduce NO concentrations (by direct and indirect NOS inhibition *via* methylarginine accumulation) without showing any negative effects of toxicity in healthy cells. For instance, it is unclear whether such dual inhibition will produce adverse effects by increasing cell toxicity by disrupting NO homeostatic levels required for normal cellular function. Alternatively, dual DDAH1/arginase inhibition is expected to inhibit NO production and simultaneously inhibit the formation of l-ornithine, the product of arginase metabolism necessary for cell proliferation/repair. This is expected to increase endogenous l-arginine concentrations, enabling NO production (*via* NOS) in non-targeted cells thus reducing cell toxicity. Therefore, dual DDAH1/arginase inhibition is proposed as be a useful strategy for selectively targeting tumor growth in multiple cancer phenotypes^11,43–45^ whilst maintaining NO homeostasis in healthy cells. More work assessing *in vivo* effects/benefits of dual DDAH1/arginase and DDAH1/NOS inhibition is needed as knowledge in this area is currently lacking.

## Conclusions and future work

Current DDAH1 inhibitors display only limited structural diversity. Most of the reported reversible *h*DDAH1 inhibitors are based on ornithine and arginine scaffolds with finite structure activity relationship (SAR) studies.^18,22,23,26,28,32^ None of these compounds inhibit DDAH1 effectively at nanomolar or picomolar concentrations. Moreover, the irreversible inhibitors have not been thoroughly investigated in human DDAH1 isoform. The only inhibitors that selectively inhibit DDAH1 and have minimal effects on arginase and nitric oxide synthase are 13 and 46. As of now, these inhibitors have not been studied in terms of metabolism and toxicity. The use of structure-based approach to discover and design DDAH1 inhibitors has been limited. More structure guided approaches utilising X-ray crystal structures and rational techniques based on *in silico* design may be needed for the discovery of structurally diverse DDAH1 inhibitors with optimal metabolic profile.

## Conflicts of interest

There are no conflicts to declare.

## Supplementary Material
